# Multianalyte nano-biosensor diagnostics: advances through microfluidic and AI integration

**DOI:** 10.3389/fbioe.2025.1715719

**Published:** 2026-01-16

**Authors:** Shashikant Pathak, Shadi Bazazordeh, Buse Çamlıca, Arnaud Delcorte, Ioulia Tzouvadaki

**Affiliations:** 1 Center for Microsystems Technology and IMEC, University of Ghent, Ghent, Belgium; 2 Institute of Condensed Matter and Nanosciences, UCLouvain, Louvain-la-Neuve, Belgium

**Keywords:** nano-biosensor, multianalyte, point-of-care, microfluidic, adaptive aritifical intelligence, wearable, electrochemical, sensor regeneration

## Abstract

Recent advances in nano-biosensors are reshaping clinical diagnostics by enabling multiplexed biomarker detection with high sensitivity and precision. This mini-review examines both the opportunities and challenges in translating nano-biosensor technologies toward clinically relevant point-of-care (PoC) and wearable devices. We emphasize the integration of multiplexing strategies with microfluidic platforms and adaptive artificial intelligence (AI) algorithms, which together enable real-time, high-throughput, and personalized health monitoring. Electrochemical and optical transduction approaches for multi-biomarker diagnostics are discussed, along with the role of microfluidic integration in enhancing sensor performance through precise sample processing, reduced reagent use, and simultaneous biomarker detection. A comparative overview of multiplexing approaches, including spatial, spectral, and temporal encoding is presented, with particular attention to sensor surface regeneration for device reusability. Furthermore, we explore the role of adaptive AI algorithms in individualising diagnostics to diverse patient groups while addressing key ethical and regulatory considerations such as algorithm transparency, patient data protection, and compliance with evolving medical device standards. By drawing together insights across nano-biosensor design, microfluidics, and AI, this mini review provides practical guidance for advancing next-generation diagnostic platforms toward clinical translation.

## 1 Introduction

Biosensors are analytical devices that detect bio-analytes (i.e., enzymes, nucleic acids, proteins, and metabolites) by converting biological interactions into measurable signals (Huang et al., 2021; Aman et al., 2025). Nano-biosensors typically incorporate nano-devices and nanomaterials (i.e., nanoparticles, nanotubes, nanowires, and nanocrystals), which offer tunable optical and electrical properties and a larger surface area (Ramesh et al., 2022). These features enhance biomolecule binding and target recognition, allowing for the accurate measurement of low-abundance targets in complex matrices (Gautam et al., 2024; Yao et al., 2014; Nasirizadeh et al., 2015; Varadharajan et al., 2025). Different types of nano-biosensors rely on distinct transduction methods. Electrochemical nano-biosensors rely on target binding events that perturb the electrical double layer (EDL) at the functionalized nano-interface, producing measurable changes in current or impedance (Wang, 2020). Optical nano-biosensors employ mechanisms such as light absorption, fluorescence, surface plasmon resonance, and refractive index modulation to achieve real-time, high-precision detection (Ahmad et al., 2023). In recent years, integrating biosensors with microfluidic platforms has advanced PoC diagnostics, enabling rapid and low-cost testing through reduced reagent use, precise sample handling, and the integration of multiple steps/assays on a single chip (Haque Ansari et al., 2016; Ch and o, 2012). This enables early disease detection and real-time monitoring of biomarkers, representing a significant advancement in diagnostic technology. Further advances in sensor regeneration, particularly when integrated into wearable biosensors (WBs), have enabled continuous, non-invasive health monitoring with enhanced stability and signal reversibility (Rosa et al., 2022; Spindel and Sapsford, 2014; Sharma et al., 2021). In addition, multiplexing functions allow simultaneous detection of multiple biomarkers, supporting comprehensive disease profiling, early diagnosis, and personalized therapy (Wei et al., 2024). The integration of adaptive and explainable AI (XAI) helps interpret complex biosensor signals, facilitate spectral deconvolution, address temporal drift, account for manufacturing variances, and optimise diagnostics (Tafadzwa Mpofu et al., 2025; Rabaï, 2024). With the increasing adherence to regulatory frameworks, these AI models provide transparent and explainable results, which are becoming increasingly important for clinical acceptance and regulatory approval.

This mini-review highlights recent advancements in nano-biosensor technologies, focusing on electrochemical and optical modalities, microfluidic integration, and emerging roles of WBs and multiplexed devices. To illustrate the evolution of the field, Section 2 begins with sensing advances that enhance signal quality, sensitivity, and stability at the single-analyte level, followed by approaches that extend these capabilities toward multianalyte detection suitable for PoC and WBs. Unlike existing reviews, which focus on isolated components, this mini-review provides a comparative outlook on multiplexing, regeneration strategies, and AI frameworks in clinical settings, as well as the regulatory and ethical aspects that tend to hinder their translation to the real world.

## 2 Nano-biosensor: electrochemical and optical sensing

### 2.1 Electrochemical sensors

**FIGURE 1:**
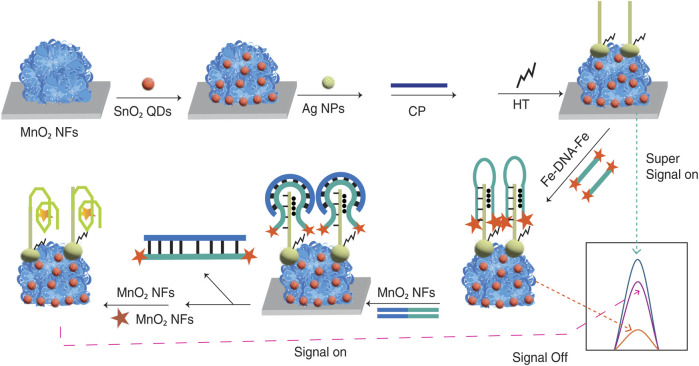
Illustration of the AgNPs/SnO_2_ QDs/MnO_2_ Nanoflowers (NFs)-based Electrochemiluminescence biosensor assembly for miRNA-21 detection. The figure depicts the stepwise construction of the biosensor, starting with the deposition of ${\text{MnO}}_{2}$ nanoflowers (NFs) on the electrode to provide structural stability and catalytic active sites. ${\text{SnO}}_{2}$ quantum dots (QDs) and Ag nanoparticles (NPs) are then layered to enhance charge transfer and provide additional catalytic sites. Capture probes (CPs) sequences are immobilized on the Ag NPs, followed by the addition of hexanethiol (HT) to prevent nonspecific bindings. Finally, double-labeled ferrocene quencher probes (Fc-DNA-Fc) are introduced to form triplex DNA structures, enabling the biosensor to achieve an “off” state with low background signal. Adapted from (Yang et al., 2021).

Electrochemical sensors enable the detection of low analyte concentrations, even achieving femtoampere-level currents or microohm-level impedance shifts, which typically requires highly optimised sensor design and instrumentation (Bertok et al., 2013; Hammond et al., 2016; Hammond et al., 2016; Zhu et al., 2015). Recent work has shown significant improvement in the sensitivity of the electrochemical sensing platform. Yang et al. achieved the limit of detection (LOD) of 0.002 fM for miRNA-21 using a hierarchical AgNPs/SnO_2_ QDs/MnO_2_ nanoflower (Figure 1), three orders below ELISA (Yang et al., 2021).

**FIGURE 2:**
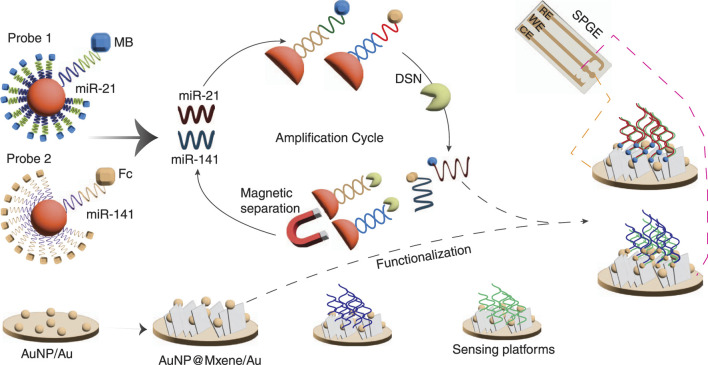
Illustration of simultaneous detection of miR-21 and miR-141 functionalized with magnetic particles (MPs) using single-stranded DNA (ssDNA) probes labelled with methylene blue (MB) and ferrocene (Fc). Upon the addition of miRNAs, duplex-specific nuclease (DSN) selectively cleaves DNA:RNA heteroduplexes, releasing uncleaved DNA sequences labeled with MB and Fc. These labelled DNA sequences are hybridized with thiolated DNA probes immobilised on the gold nanoparticle (AuNP@MXene/Au), enabling the detection of miR-21 and miR-141. Adapted from (Shin Low et al., 2021).

Similarly, gold nanoparticles reduced graphene oxide (AuNP-rGO) electrodes exploit graphene’s $2x10^{5}$ ${\text{cm}}^{2}$ /V-s mobility and thiol-selective AuNP anchors to measure the LOD of 0.0319 fM for miR-141 directly in patient plasma (Yu et al., 2025; Shin Low et al., 2021; Chandra Barman et al., 2024). The integration of new materials such as MXene- ${\text{Ti}}_{3}{\text{C}}_{2}$ Tx modified with 5 nm AuNPs electrodes were used to detect miRNA-21 and miRNA-141 (Figure 2), respectively, with synergetic signal amplification, achieving sensitivities of 204 aM and 138 aM, across a wide linear range (500 nM–50 nM) (Mohammadniaei et al., 2020).

Clinical validation studies demonstrate the translational readiness of these platforms. CRISPR-Cas12a (Clustered Regularly Interspaced Short Palindromic Repeats CRISPR associated protein 12a) biosensors achieved 96.5% sensitivity and 99% specificity for Severe Acute Respiratory Syndrome Coronavirus 2 (SARS-CoV-2) detection in saliva (n = 352 patients) relative to Reverse Transcription quantitative Polymerase Chain Reaction (RT-qPCR), showcasing the diagnostic potential of integrated biosensing approaches (Abugattas-Núñez del Prado et al., 2024).

### 2.2 Optical sensors

**FIGURE 3:**
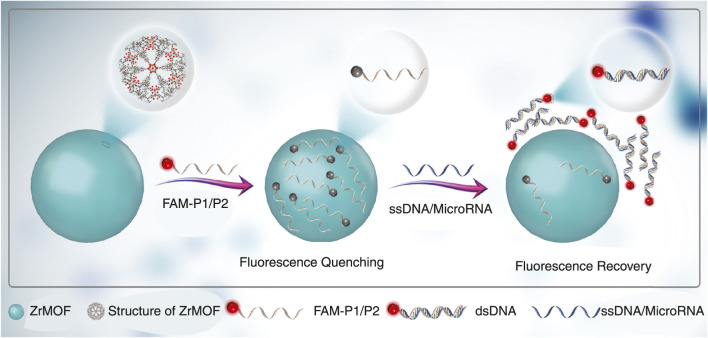
Schematic illustration of the switch fluorescence sensing platform for DNA and miRNA detection using ZrMOF and FAM-labeled probes, which serve as effective fluorescence quenchers. Adapted from (Liu et al., 2022).

**FIGURE 4:**
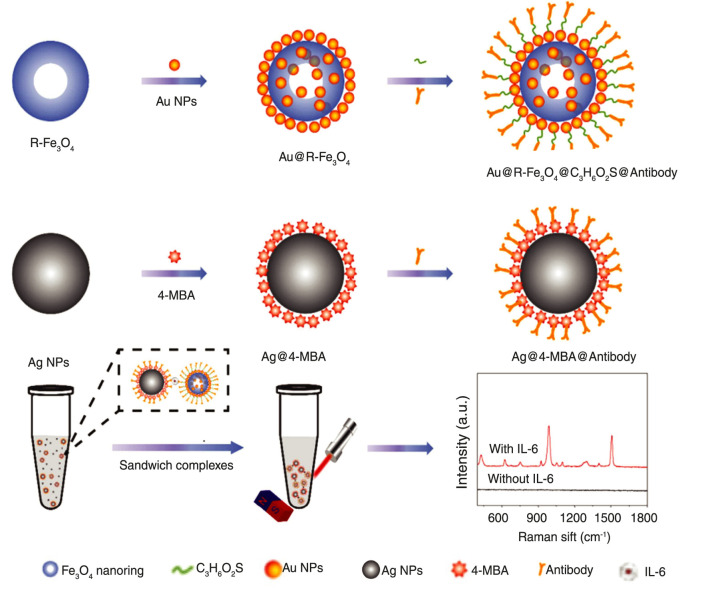
Scheme of IL-6 detection procedure by the magnetic SERS immunoassay based on R- ${\text{Fe}}_{3}{\text{O}}_{4}$ . Silver nanoparticles (Ag NPs) modified with 4-mercaptobenzoic acid (4-MBA) and antibodies serve as the probe, while gold nanoparticles (Au NPs) and specific antibody-functionalized R- ${\text{Fe}}_{3}{\text{O}}_{4}$ act as the capture substrate. Upon addition of the target molecule, it will be coupled with an antibody to form a sandwich-like structure. The detection range is 0.1–1,000 pg/mL, and the limit of detection is 0.028 pg/mL. Adapted from (Xie et al., 2023).

Optical nano-biosensors detect analyte-receptor interactions through light absorption, fluorescence, surface plasmon resonance, and refractive index changes, offering real-time and highly sensitive detection for clinical and PoC applications (Chen and Wang, 2020; Mostufa et al., 2024). Liu et al. designed a zirconium porphyrin metal-organic framework switch-type fluorescence biosensor for ssDNA and microRNA-21 using fluorescence resonance energy transfer and photo-induced electron transfer, achieving detection limits of two fM and 11 aM within 30 min, without complex immobilization, and effective in human serum (Figure 3) (Liu et al., 2022; Ya et al., 2024). Xi et al. developed a surface-enhanced Raman scattering (SERS) (Figure 4) sandwich immunoassay with ${\text{Fe}}_{3}{\text{O}}_{4}$ nanorings for interleukin-6 (IL-6) detection, achieving 0.028 pg/mL (Xie et al., 2023; Vairaperumal et al., 2023; Lee et al., 2024). Ming-Kiu et al. developed a heterogeneous optical assay for Ebola viral genes using ${\text{BaGdF}}_{5}$ :Yb/Er upconversion nanoparticles with oligonucleotide probes and gold nanoparticles on a nanoporous anodic alumina membrane, achieving 50–700 fM detection (Tsang et al., 2016; Song et al., 2021).

**FIGURE 5:**
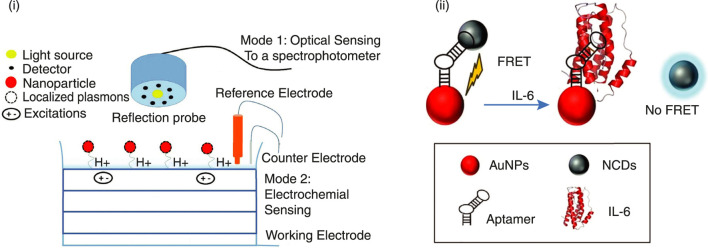
**i)** Scheme for the dual-mode biosensor: Mode 1 is the optical sensing, where the reflection probe shows an integrated light source and detector to measure light absorbance from the surface. Mode 2 shows an electrochemical sensing setup with a conventional 3-electrode setup to measure the capacitance of the electrolyte–insulator–semiconductor structure. Reprinted from (Bhalla and Estrela, 2018). **(ii)** A label-free and specific FRET-based IL-6 aptasensor was developed using a DNA aptamer modified with nitrogen-doped carbon quantum dots (NCDs) and AuNPs as a donor-quencher pair. Reproduced with permission from Springer Nature (Mahani et al., 2022).

Mahani et al. presented an ultrasensitive fluorescence resonance energy transfer aptasensor for IL-6 using nitrogen-doped carbon quantum dots and gold nanoparticles as a donor-quencher pair, with a detection limit of 0.82 pg/mL (S/N = 3) (Figure 5(ii)) (Mahani et al., 2022). Bhalla et al. demonstrated a dual-mode electro-optical biosensor using localized surface plasmons in gold nanoparticles for kinase inhibitor screening, showing potential for versatile bio/chemical sensing (Figure 5(i)) (Bhalla and Estrela, 2018).

## 3 Diagnostic applications

### 3.1 PoC multianalytes

Nano-enabled PoC diagnostics are advancing from single to multi-analyte detection. Clinically useful panels require distinguishing several targets in one sample without signal interference. Orthogonal encoding assigns each analyte a unique signal dimension (spatial location, spectral signature, or temporal phase) (Lu et al., 2020), enabling simultaneous detection with very high sensitivity in the aM–pM range.

**FIGURE 6:**
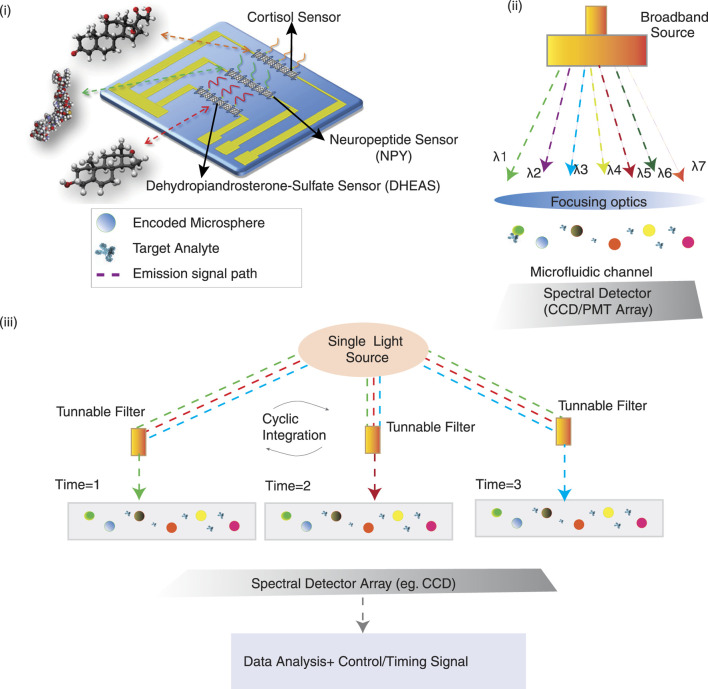
(i) DNA strands wrap around single-walled carbon nanotubes (SWCNTs) to enable the attachment of multiple aptamers specific to cortisol (orange), NPY (green), and DHEAS (red) adapted from (Xu et al., 2018). (ii) Schematic of a broadband excitation and spectral detection system integrated with a microfluidic channel. (iii) Schematic of a time-resolved multiplex assay using a single light source and tunable filters for cyclic spectral integration and analysis.

Spatial multiplexing utilises nanostructured microelectrode arrays and microfluidic compartmentalization to provide separate sensor sites for each analyte (Figure 6(i)). O’Brien et al. employed this strategy through electrodeposition of Au nanostructures from MXene-based materials, achieving 0.04–0.5 pg/mL detection for breast cancer markers HER-2 (human epidermal growth factor receptor 2), MUC-1 (Mucin 1), and CA15-3 (Cancer antigen 15–3) (Brien et al., 2024).

Spectral multiplexing allows simultaneous multiple analyte (Cortisol, NPY, DHEAS) readouts by spectral deconvolution using nanomaterial-based optical barcoding systems (Figure 6(ii)) such as quantum dot-labelled antibodies or plasmonic nanoparticles (Hu et al., 2018; Klostranec et al., 2007; University of Toronto-Division of the Vice-President and Research and Innovation, 2019). The Luminex xMAP (Luminex Multi-Analyte Profiling) platform uniquely uses barcoded microspheres conjugated with capture antibodies to resolve >30 immune analytes at the pg level for neonatal sepsis diagnostics (Li et al., 2025).

Another strategy involves the temporal multiplexing (Figure 6(iii)), which allows the reversible sensor surface functionalization and cyclic electrochemical interrogation using antifouling chemistries like zwitterionic polymers and nano-engineered self-cleaning electrodes (Zhou et al., 2024).

Lu et al. reported electrochemical biosensors achieving >95 % signal retention across multiple reuse cycles with minimal cross-contamination (Lu et al., 2025). The next frontier of multiplexed diagnostics is hybrid platforms that combine spatial, spectral, and temporal encoding strategies, enhanced with AI-based signal deconvolution algorithms, to maximise signal extraction, reduce crosstalk, and accommodate non-linear sensor behaviour for robust real-world multi-biomarker diagnostics (Han et al., 2025).

### 3.2 Wearable multianalyte biosensors and regeneration

Beyond the challenges associated with PoC sensing, WBs introduce additional demands and requirements that must be addressed to ensure optimized functionality, reliable biosensing capability, and long-term stability. These compact systems integrate with the body through tattoos, textiles, or implants to monitor biomarkers continuously, but they often face issues with stability and signal recovery. Regenerative sensing methods using electrochemical or optical cleaning can reduce cost and enable continuous personalized monitoring (Jia et al., 2024).

**FIGURE 7:**
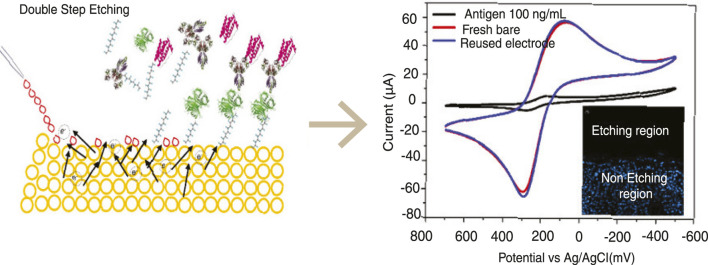
Double-step etching approach for electrode regeneration in a wearable sensor platform. (Left) Schematic illustration of the surface process enabling antigen removal and electrode reuse. (Right) Representative cyclic voltammetry curves comparing fresh, reused, and antigen-bound electrodes, with an inset highlighting etched and non-etched regions. Adapted from (Lee et al., 2025).

Electrochemical regeneration strategies have shown particular promise for extending sensor lifetimes. Lee et al. demonstrated that cyclic voltammetric sweeps in ferri/ferrocyanide restore screen-printed gold electrodes (SPGEs) through electrochemical cleaning pulses, enabling multiple reuse cycles (Lee et al., 2025) (Figure 7).

Choi et al. introduced an *in-situ* electrochemical regeneration method for microfluidic biosensors using low-voltage desorption of short-chain self-assembled monolayers (SAMs) on Au electrodes. The approach enabled up to 50 reuse cycles with relative standard deviation (RSD) below 0.82%, demonstrating stable performance (Choi and Chae, 2009). Liu et al. proposed an optofluidic surface-enhanced Raman scattering (SERS) sensor with photothermal-assisted regeneration, where light-induced heating removes bound analytes. The device achieved an LOD of $10^{-13}$ mol/L for Rhodamine 6G and shows promise for reusable, WBs or PoC platforms (Liu et al., 2021). Furthermore, Shin et al. developed a microfluidic impedance biosensor for automated monitoring of cell-secreted biomarkers, detecting albumin and glutathione-S-transferase-alpha (GST- α ) at ng/m levels. Using electrochemical cleaning, the platform enabled up to 25 regeneration cycles without loss of sensitivity, supporting long-term organoid-based drug screening (Shin et al., 2017).

### 3.3 Microfluidic integration

**FIGURE 8:**
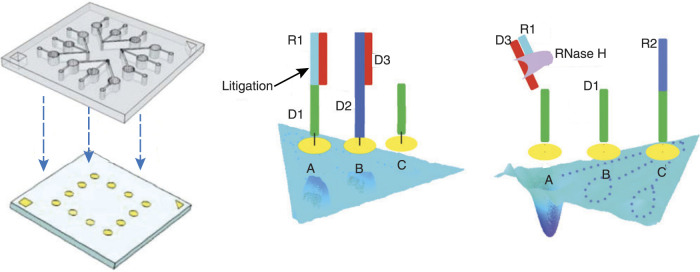
A four-chamber microfluidic biochip is fabricated for the rapid detection of multiple proteins and nucleic acids. Adapted from (Hu Seefeld et al., 2011).

Integrating microfluidics with nanotechnology enables sensitive, specific, real-time biomarker detection from very small samples. Precise control of fluid flow and reaction conditions reduces variability, limits sample loss, and improves signal quality, resulting in more accurate and reliable biosensor measurements (Liao et al., 2019; Dong et al., 2019; Sekhwama et al., 2024; Bhalla et al., 2020). Seefeld et al. developed a microliter-volume surface plasmon resonance imaging biochip featuring a 12-element microarray embedded within Polydimethylsiloxane (PDMS) microfluidic chambers. This platform achieved remarkable sensitivity, detecting ssDNA at concentrations as low as 1 fM ( ∼18,000 molecules) in under 200 s, facilitated by RNase H-based amplification (Figure 8) (Hu Seefeld et al., 2011).

**FIGURE 9:**
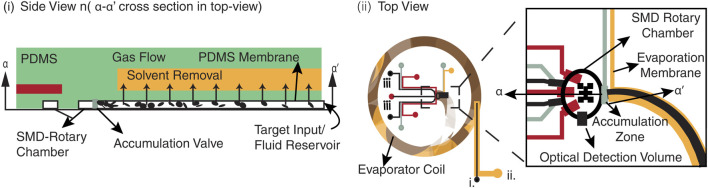
Schematic of the combined microevaporator/rotary SMD microdevice. **(i)** Side view of the operating micro-evaporator, prior to sample transfer into the detection chamber. Solvent removal through the pervaporation membrane must be compensated by convection from the sample reservoir, while actuation of the accumulation valve enables target collection at the dead end. **(ii)** The control layer (lighter grey) shows the evaporation membrane, rotary pump, and isolation valves. Target accumulation is accomplished by solvent removal from the fluidic layer (black, inlet labelled i.) through the pervaporation membrane (inlet labelled ii.). Following target accumulation, the concentrated sample plug is transferred to the SMD rotary chamber for probe hybridisation and detection; probes and hybridisation buffer are introduced through separate inlets (labelled iii.). Adapted from (Puleo and Wang, 2009).

The first integration of nanofabricated memristive biosensors with a microfluidic platform was reported by Vallero et al. (2016). The design incorporates tailored metal interconnects to preserve the electrical readout of the memristive nanowire sensors. The microfluidic system enhances assay performance by enabling controlled washing surface biofunctionalization and subsequent analyte-exposure steps. Additionally, Dallari et al. developed a rapid, versatile, low-cost SERS-microfluidic platform, coupled with a portable fiber-based Raman setup for detection of amyloid- β 1–42 (A β ), demonstrating feasibility for Alzheimer’s diagnostics (Dallari et al., 2020). Puleo et al. further advanced microfluidic capabilities by integrating a microfluidic system with inline micro-evaporators to concentrate DNA targets into nanoliter single-molecule fluorescence detection chamber, enabling molecular beacon hybridization and detection from initial DNA concentrations of 50 aM (Figure 9) (Puleo and Wang, 2009).

Moreover, Chan et al. developed a diagnostic system integrating QDs and microfluidics to enable multiplexed, high-throughput detection of serum biomarkers for major blood-borne infectious diseases. The platform requires less than 100 μ L of sample, provides results in under an hour, achieves approximately ( $10^{-10}$ – $10^{-12}$ )M sensitivity, and allows precise simultaneous measurements with minimal cross-reactivity (Klostranec et al., 2007).

### 3.4 AI integration

An adaptive AI algorithm is essential for extracting diagnostic information from PoC multi-analyte and WBs. The complex, high-dimensional signals generated by nano-biosensors, including overlapping spectral signatures from multiple analytes, time-dependent sensor drift, and batch-to-batch fabrication variability, exceed the capabilities of conventional signal processing methods (Park et al., 2024). Therefore, selecting appropriate AI architectures such as CNN (convolutional neural network), LSTM (Long Short-Term Memory), transfer learning, or ensemble methods is critical to address these challenges.

CNNs are particularly suited for analyzing multi-analyte biosensors, as they separate overlapping signals from different biomarkers. Li et al. demonstrated CNN-based spectral unmixing with a Root Mean Square Error (RMSE) of approximately $6.42x10^{-2}$ using 160,000 training samples (Li et al., 2022). This is essential when multiple analytes produce interfering signals in the same sensor.

However, biosensors also face temporal challenges as sensor signals drift over time due to degradation, temperature changes, and calibration loss. LSTM networks address this by learning temporal patterns in biosensor data, maintaining accuracy during continuous monitoring in WBs. Recent studies showed LSTM models achieved high accuracy (i.e., MSE (Mean Squared Error) = 0.124, R^2^ (R-squared score) = 0.945) in forecasting and anomaly detection tasks (Ishfaque et al., 2022), with benchmarks confirming that LSTM and hybrid RNN (recurrent neural network) models consistently deliver lower error rates (i.e., MAE (Mean Absolute Error): 2.96, MAPE (Mean Absolute Percentage Error): 7.45%, RMSE: 4.04) and greater robustness compared to traditional RNNs (Yunita et al., 2025).

Transfer learning addresses manufacturing variability by enabling models pre-trained on large datasets to be fine-tuned for specific sensor batches with limited data. Zhang et al. demonstrated that this approach can reduce calibration needs by 50% without compromising performance (Wang et al., 2025).

The adoption of XAI enhances clinical adoption by making AI decision-making transparent and interpretable. XAI methods reveal how models arrive at predictions, crucial for regulatory approval and clinician trust (Nabee and Hugh Li, 2025; Vaiyapuri, 2024). Techniques such as SHAP and saliency maps identify key features influencing diagnostics, helping clinicians validate biomarkers and understand prediction rationale (Sathyan et al., 2022; Lopes et al., 2023). XAI also aids in identifying sensor drift and guiding recalibration. While each AI method addresses specific challenges, real-world diagnostic systems often face multiple issues simultaneously. Ensemble methods combine multiple AI models to leverage complementary strengths and improve robustness across diverse sensor conditions and patient populations (Vaiyapuri, 2024; Ishfaque et al., 2022).

## 4 Ethical challenges of using AI

AI integration in nano-biosensor diagnostics faces critical ethical challenges with unique implications for sensor-based platforms. These AI systems directly inform clinical decisions, making ethical considerations fundamental to their validity and clinical utility. However, significant challenges persist. Algorithm bias, for instance, remains a significant problem with only 3.6% of 903 FDA (Food and Drug Administration)-cleared AI systems validated across racial/ethnic groups and 0.9% included socioeconomic diversity, producing systematically lower accuracy for underrepresented populations (Muralidharan et al., 2024; Windecker et al., 2025).

In nano-biosensors, this bias is amplified by physiological variations. Optical sensors exhibit different performance across skin tones due to melanin absorption, while electrochemical sensors show varied responses to sweat chemistry and pH differences across ethnic groups, yet these variations are rarely represented in training datasets.

Lack of interpretability in deep learning models prevents clinicians from distinguishing whether signal changes reflect true biomarker variations or sensor artefacts (fouling, drift, temperature effects), creating diagnostic ambiguity when AI misinterprets degraded sensor responses as clinically meaningful patterns (Nabee and Hugh Li, 2025). Privacy risks arise from continuous wearable monitoring, where temporal biosensing patterns combined with activity and location metadata enable patient re-identification despite anonymization (Cross et al., 2024; Williamson and Prybutok, 2024).

Regulatory frameworks address these concerns variably. The FDA mandates pre-market clinical validation and post-market surveillance for adaptive algorithms (European Medicines A gency EMA, 2023). The European Union (EU) AI Act classifies diagnostic AI as high risk, requiring algorithm traceability, bias audits, and public model documentation (European Parliament, 2023). China’s National Medical Products Administration (NMPA) enforces demographic representation in clinical trials and data localization under the Personal Information Protection Law (PIPL) (Cross et al., 2024). Health Canada aligns with FDA standards while emphasizing transparency (Onitiu et al., 2024). Divergent requirements hinder global deployment.

XAI methods provide mechanistic insights into algorithmic decision-making (Zhang et al., 2025), while federated learning architectures with differential privacy achieve 90% reduction in re-identification risk across regulatory compliances such as Health Insurance Portability and Accountability Act (HIPAA), General Data Protection Regulation (GDPR), and PIPL (Cross et al., 2024; Williamson and Prybutok, 2024; Guru, 2023). Yet these cannot compensate for fundamentally unrepresentative datasets or regulatory fragmentation. Clinical translation requires integrating transparency, bias mitigation, and privacy protection as foundational design principles alongside international regulatory harmonization for equitable global access.

## 5 Challenges and future directions

### 5.1 Challenges

Variations in temperature, mechanical strain, and biofluid complexity compromise sensor stability, causing signal attenuation and reduced batch-to-batch reproducibility by altering surface energetics (Gulati et al., 2025; Luo et al., 2024; Jia et al., 2024; Preetam et al., 2022). On flexible substrates and nanomaterial electrodes, mechanical stress induces delamination, microcracking, and signal drift that degrade sensitivity and reproducibility (Kim et al., 2019; Kim et al., 2024). In optical systems, nanoscale structural deviations shift plasmonic resonance frequencies, creating response heterogeneity that compounds protein adhesion-induced signal loss (Adhi Prabowo et al., 2021; Kulkarni et al., 2022; Pandit et al., 2016). Sensor regeneration strategies demonstrate feasibility over limited cycles under benign conditions but lack validation under prolonged physiological fouling essential for practical wearable deployment. In PoC and WBs, micro-liter-scale sample volumes increase susceptibility to evaporation and contamination.

AI integration faces complex technical and ethical challenges. Edge computing memory restrictions (kilobytes to megabytes) cannot accommodate CNNs required for complex biological matrix analysis, while interpretability mandates exceed available processing power (Park et al., 2024; Wang et al., 2025). Training dataset limitations with insufficient demographic diversity create algorithmic bias incompatible with equitable deployment (Vaiyapuri, 2024), manifesting as differential optical responses across skin tones and electrochemical variations in sweat chemistry across ethnic groups. Black-box AI architectures obscure whether signal changes reflect true biomarker variations or sensor artefacts, preventing clinical validation of model outputs.

Inadequate validation hinders clinical translation. Performance is often evaluated in spiked buffer systems under controlled conditions rather than complex biological matrices containing interfering substances, variable pH, and protein fouling, which degrade sensor performance and reproducibility in clinical environments. This validation gap explains frequent disparities between reported laboratory metrics and actual clinical utility.

Regulatory frameworks compound these obstacles through contradictory requirements and fragmentation. Current FDA-cleared systems demonstrate severe validation gaps (3.6% racial/ethnic, 0.9% socioeconomic representation) (Muralidharan et al., 2024; Windecker et al., 2025). EU AI Act traceability mandates require comprehensive algorithm documentation scaling exponentially with system complexity, while harmonizing international regulatory standards for adaptive algorithms remains unresolved (European Parliament, 2023; European Medicines A gency EMA, 2023; Onitiu et al., 2024). These challenges reveal that incremental improvements to individual sensor components cannot address systemic barriers to clinical translation.

### 5.2 Future directions

The path forward requires integrated approaches spanning multiple disciplines. Addressing sensor material and physical limitations requires advances in surface chemistry and detection mechanisms. Zwitterionic hydrogel coatings demonstrate significant improvements in antifouling properties and long-term signal fidelity (Wen et al., 2024; Zhou et al., 2024; Bakr et al., 2025; Yang et al., 2023), while self-calibrating architectures and nano-engineered surface chemistries show promise for mitigating signal drift and batch-to-batch variability (Jia et al., 2024). Quantum sensing represents an emerging frontier with potential to surpass current sensitivity-stability trade-offs. Marie et al. demonstrated label-free, real-time biomolecule detection with enhanced spatial resolution (Krečmarová et al., 2021), and Zalieckas et al. achieved 10 pM detection for mRNAs (Zalieckas et al., 2024), suggesting pathways toward single-molecule sensitivity in physiologically relevant environments.

Validation standards must evolve beyond proof-of-concept demonstrations to systematic assessment in authentic patient samples across diverse demographics and complex biofluids. AI model development must incorporate bias mitigation and interpretability as foundational design principles, with training datasets deliberately constructed to represent physiological and demographic diversity. Regeneration and anti-fouling technologies require validation across extended reuse cycles under realistic fouling conditions. Multiplexing strategies must demonstrate robust performance for clinically relevant biomarker panels with validated diagnostic utility. Regulatory frameworks across the US FDA, EU, China, and Health Canada continue evolving toward risk-based, lifecycle-oriented approaches for AI-enabled medical devices (European Medicines A gency EMA, 2023; European Parliament, 2023; Onitiu et al., 2024; Cross et al., 2024). However, quantum sensing platforms pose distinct regulatory challenges not addressed in current guidance (Kop et al., 2025).

Current frameworks lack quantum-specific evaluation protocols for assessing superposition-based measurements and entanglement-enhanced sensitivity (Fairbairn et al., 2025). Quantum computing advances threaten existing RSA (Rivest-Shamir-Adleman) encryption, posing critical data security challenges for patient health information and medical device integrity (Kop et al., 2025). Addressing these quantum-specific regulatory gaps requires establishing specialised evaluation frameworks that recognize quantum mechanical principles while ensuring patient safety and data privacy (Fairbairn et al., 2025; Kop et al., 2025). International harmonization remains essential to reduce compliance burdens while maintaining safety standards and enabling equitable global access.

The convergence of nanomaterials, microfluidics, multiplexing, and AI offers powerful potential for continuous, personalized health monitoring. Achieving this requires shifting from optimizing components in isolation to designing integrated, clinically robust systems that perform reliably and equitably in real-world settings. Clinical translation will depend on demonstrating improved diagnostic accuracy, accessibility, and health outcomes across diverse populations. Ultimately, sustained interdisciplinary collaboration will determine whether nano-biosensor diagnostics fulfill their promise of accessible, accurate, and actionable health monitoring worldwide.
