# Correction: Multianalyte nano-biosensor diagnostics: advances through microfluidic and AI integration

**DOI:** 10.3389/fbioe.2026.1855897

**Published:** 2026-06-08

**Authors:** Shashikant Pathak, Shadi Bazazordeh, Buse Çamlıca, Arnaud Delcorte, Ioulia Tzouvadaki

**Affiliations:** 1 Center for Microsystems Technology and IMEC, University of Ghent, Ghent, Belgium; 2 Institute of Condensed Matter and Nanosciences, UCLouvain, Louvain-la-Neuve, Belgium

**Keywords:** nano-biosensor, multianalyte, point-of-care, microfluidic, adaptive aritifical intelligence, wearable, electrochemical, sensor regeneration

Affiliation “Institute of Condensed Matter and Nanosciences, UCLouvain, Louvain-la-Neuve, Belgium” was omitted for author Shadi Bazazordeh. This **affiliation** has now been added for author Shadi Bazazordeh.

Author **Arnaud Delcorte** was omitted as an author in the published article. The correct author list reads:

Shashikant Pathak^1^, Shadi Bazazordeh^1,2^, Buse Çamlıca^1^, Arnaud Delcorte^2^ and Ioulia Tzouvadaki^1^

The **Author Contributions** Statement has been corrected to read:

SP: Writing – original draft, Writing – review and editing. SB: Writing – original draft, Writing – review and editing. BÇ: Writing – original draft, Writing – review and editing. AD: Supervision, Writing, review and editing. IT: Writing – review and editing.

The original article has been updated.

